# Phylogeny, Functional Annotation, and Protein Interaction Network Analyses of the *Xenopus tropicalis* Basic Helix-Loop-Helix Transcription Factors

**DOI:** 10.1155/2013/145037

**Published:** 2013-11-10

**Authors:** Wuyi Liu, Deyu Chen

**Affiliations:** Department of Biology Science, Fuyang Normal College, No. 100 West Qing He Road, Fuyang 236037, China

## Abstract

The previous survey identified 70 basic helix-loop-helix (bHLH) proteins, but it was proved to be incomplete, and the functional information and regulatory networks of frog bHLH transcription factors were not fully known. Therefore, we conducted an updated genome-wide survey in the *Xenopus tropicalis* genome project databases and identified 105 bHLH sequences. Among the retrieved 105 sequences, phylogenetic analyses revealed that 103 bHLH proteins belonged to 43 families or subfamilies with 46, 26, 11, 3, 15, and 4 members in the corresponding supergroups. Next, gene ontology (GO) enrichment analyses showed 65 significant GO annotations of biological processes and molecular functions and KEGG pathways counted in frequency. To explore the functional pathways, regulatory gene networks, and/or related gene groups coding for *Xenopus tropicalis* bHLH proteins, the identified bHLH genes were put into the databases KOBAS and STRING to get the signaling information of pathways and protein interaction networks according to available public databases and known protein interactions. From the genome annotation and pathway analysis using KOBAS, we identified 16 pathways in the *Xenopus tropicalis* genome. From the STRING interaction analysis, 68 hub proteins were identified, and many hub proteins created a tight network or a functional module within the protein families.

## 1. Introduction

Transcription factors are usually classified into different families based on their sequence of functional DNA-binding or protein-binding domains, which are highly conserved among many species and include many members mediating cell fate allocation during animal and plant development [1–11]. The expression and activity of basic helix-loop-helix (bHLH) transcription factors can be regulated in response to cell-cell signaling, leading to the transcription of specific sets of genes required for a cell to adopt particular fates. Due to their important functions found in various organisms, bHLH transcription factors have been the subject of many researches. The first report of bHLH transcription factors focused on the murine factors E12 and E47 [12]. Later, more and more bHLH proteins have been identified in living organisms. In 1997, Atchley and Fitch [1] proposed an organization for the classification of the bHLH proteins based on the phylogenetic analysis of the 122 bHLH domains combined with the presence or absence of another additional domain. Their analysis allowed for the defining of four different groups of bHLH protein families according to structural similarities [1]. This classification was performed using only the bHLH motif or domain, because the flanking regions for bHLH proteins are very divergent. Atchley and Fitch's classification led to the postulation of four distinct groups based on amino acid patterns and E-box-binding specificity [1]. In 2002, Ledent et al. [4] defined 44 orthologous families or sub-families and 6 supergroups based on the DNA-binding activities of bHLH transcription factors after large-scale phylogenetic analyses. After the revision of Simionato et al. [6] in 2007, animal bHLH proteins are reclassified into 45 families. Among these 6 supergroups, members of groups A and B are common bHLH proteins [1, 3–6]. Group A proteins bind to CACCTG or CAGCTG, while group B proteins bind to CACGTG or CATGTTG. The consensus DNA binding sequences for these bHLH proteins form the typical E boxes (CANNTG). Group C proteins are complex molecules with one or two PAS domains following the bHLH domain, being inclined to bind the core sequence ACGTG or GCGTG. They are mainly responsible for the regulation of midline and tracheal development, circadian rhythms, and gene transcription in response to environmental toxins. Group D proteins correspond to bHLH proteins that are unable to bind to DNA due to lack of a basic domain. Both, group D and group F, are proteins that lack basic parts and act as antagonist partners of group A proteins in the heterodimers. Particularly, group F are a kind of COE proteins characterized by the presence of an additional COE domain involved in both dimerization and DNA binding. Group E proteins are another type of special transcription factors. They usually contain two additional domains named “Orange” and “WRPW” peptides in their carboxyl termini and they bind preferentially to sequences typical of N boxes (CACGCG or CACGAG). Generally speaking, all of the bHLH transcription factors share a common bHLH motif or domain of approximately 60 amino acids, which contains a basic region and two helices separated by a loop (HLH) region of variable length [3–5, 12]. The basic region is a DNA-binding domain, and the amphipathic *α*-helices of two bHLH proteins can interact with each other. The HLH domain promotes dimerization, and interaction between the helix regions of two different bHLH proteins leads to the formation of homodimeric or heterodimeric complexes, while the basic region of each partner recognizes and binds to a core hexanucleotide DNA sequence [2–4]. In a couple of reports [13, 14], Atchley et al. inferred a predictive motif for the bHLH domains based on 242 bHLH proteins, in which 19 conserved sites were found within the bHLH domains. It was found and proved that a sequence with no more than 9 mismatches could be a putative bHLH protein [15].

Recently, in many organisms whose genomes have been released and are available, more and more bHLH proteins have been identified and bHLH transcription factor families have been analyzed due to their important and pivotal regulatory functions displayed in various organisms [3–25]. As well as *Xenopus laevis* the *Xenopus tropicalis* is a model organism for researches testing the developmental, behavioral, and neurological consequences of genetic variation [26–28]. The draft of *Xenopus tropicalis* genome assembly was submitted by American scientists at the Lawrence Berkeley National Laboratory in California [28], and the *Xenopus tropicalis* genome project is still underway. In previous work, the preliminary survey identified 70 bHLH transcription factors [16]. Recently, we found it was incomplete and the functional properties and regulatory networks of bHLH transcription factors were not fully analyzed. In this study, we used the criteria developed by Atchley et al. [13] and the 45 representative bHLH domains defined by Ledent et al. [4] and Simionato et al. [6] to do updated searches using BLAST search algorithms in the *Xenopus tropicalis* genomic database and identified 105 bHLH proteins. We next made large-scale phylogenetic analyses of the *Xenopus tropicalis* bHLH domains with the 118 human bHLH domains [6]; this allowed us to define the full set of bHLH orthologous genes and their related families. We further report the result of analyses of gene ontology (GO) annotations, functional pathways, and protein interaction networks based on the *Xenopus tropicalis* genomic databases.

## 2. Materials and Methods

### 2.1. BLAST Searches and Retrieval of bHLH Domains

At first, we followed the criteria developed by Atchley et al. [1, 13] to define a bHLH protein [13]. These searches initially yielded a few bHLH transcription factors (up to 20 protein sequences). The deduced predictive protein consensus motif of Atchley et al. [13] is “++X_(3–6)_E + XRX_(3)_ *α*NX_(2)_ΦX_(2)_L + X_(5–22)_ + X_(2)_KX_(2)_ *δ*LX_(2)_A*δ*XY*α*X_(2)_L” where + = K, R; *α* = I, L, V; Φ = F, I, L; *δ* = I, V, T; E, R, K, A, and Y are as defined; X = any residue; X_(*i*)_ = any *i* residues; and X_(*i*–*j*)_ = *i* to *j* of any residues. We also used the 45 representative bHLH domains from the tables provided by Ledent et al. [4] and Simionato et al. [6] to make multiple TBLASTN and BLASTP searches of bHLH domains against the *Xenopus* Genome Resources built by NCBI (http://www.ncbi.nlm.nih.gov/genome/guide/frog/) and Xenbase (http://www.xenbase.org/) for all putative bHLH proteins. Then, PSI-BLAST searches were conducted against the nonredundant database of *Xenopus* genomes at NCBI using the representative bHLH domain sequences. All of the TBLASTN, BLASTP, and PSI-BLAST searches were conducted with the methods and similar parameter setting-ups in the previous works [7, 16]. With these BLAST searches above, we obtained all of the putative bHLH proteins with no more than 9 mismatches among the 19 amino acids residues [15]. Moreover, we also did TBLASTN searches of frog EST data against the *Xenopus *Genome EST databases with a stringency set as *E* ≤ 0.0001 and an identity of 90% or higher as candidacy. The obtained EST data were translated into protein sequences using online analysis tools (http://www.genoscope.cns.fr/agc/tools/) to verify the putative bHLH sequences found.

### 2.2. Manual Improvement and Sequence Alignment

Protein sequence accession numbers and genomic contig numbers were finally obtained by BLASTP and TBLASTN searches against the *Xenopus tropicalis* protein databases and genome sequence assembly (reference only) with the amino acid sequence of each identified bHLH domain. All of the obtained sequences were aligned using ClustalX 2.0 [29]. Redundant sequences of candidates were removed according to their corresponding serial numbers of the scaffold or clone or genomic contig, gene ID, protein ID, coding region, and alignment information. The, finaly, aligned bHLH domains were shaded using GeneDoc 2.6.02 [30] and copied into an RTF file for further annotation.

### 2.3. Analyses of Gene Ontology (GO) Annotations and Pathways

A functional annotation analysis of *Xenopus tropicalis* bHLH transcription factor genes was conducted. Gene ontology (GO) function enrichment was analyzed using DAVID Functional Annotation Bioinformatics Tools [31, 32], which use the ontology hierarchy tree and calculates and report statistical significance for GO term categories with a hypergeometric *P* value and enrichment scores. This approach directly scores predefined gene sets and/or pathways based on given gene lists.

All of the bHLH transcription factor genes were also subjected to KOBAS analysis (http://kobas.cbi.pku.edu.cn/home.do), and significant pathways were retrieved at the default *P*  value ≤ 0.5. We applied KOBAS vocabulary to first annotate all genes with corresponding KO and then identify both, the most frequent the statistically significantly enriched pathways. With rather strict cutoff of FDR ≤ 0.05, KOBAS found statistically significantly enriched pathways, as shown in Table 3.

We could thus identify and select significantly enriched gene ontology terms and pathways using bioinformatics databases DAVID [31, 32] and KOBAS [33–35], respectively. We selected the functional categories that were more likely to be biologically meaningful by statistical significance of each functional category in the input set of genes versus all annotated genes in the *Xenopus tropicalis* genome.

### 2.4. Protein Interaction Network Analysis

To investigate possible interactions between the gene lists from our updated surveys, the STRING search tool was used for the creation of protein interaction network (PIN) files as previously described [36–38]. To increase the completeness of our results, this search was set to include full information extracted from the STRING biological interaction databases. The created networks were explored and compared based on their topological characteristics and gene products (proteins) by default with a confidence of score 0.15 [38].

### 2.5. Phylogenetic Analyses

The putative *Xenopus tropicalis* bHLH protein sequences, together with the human bHLH domains, were used to construct phylogenetic trees based on bayesian inference (BI) by MRBAYES 3.1.2 [37, 38] and maximum likelihood estimation (MLE) by PHYML 2.4.4 [44] with the JTT substitution frequency matrix [45], respectively. Phylogenetic analyses by BI and MLE were performed with the methods and similar parameter setting-ups in the previous works [7, 16]. Briefly, the BI analysis was performed with two independent Markov chains, each containing from 800 to 1100 million Monte Carlo steps until the standard deviation of split frequencies was below 0.01, with sample frequency saved every 1000 generations. Finally, all of the obtained trees were edited and displayed by means of the software package MEGA 4.0 [46]. 

## 3. Results and Discussion

### 3.1. Data Retrieval and Identification of bHLH Transcription Factors

The names and related information of the putative *Xenopus tropicalis* bHLH proteins are listed in Table 1. All of the bHLH domains obtained had more than 10 conserved amino acids [15]. The putative bHLH proteins were named according to their phylogenetic relationship with its corresponding human orthologs and paralogs. If a human bHLH sequence had two or more *Xenopus tropicalis* orthologous genes, we used “a,” “b,” and “c” or “1,” “2,” and “3” and so on, to number them. In the present work, 34 frog hypothetical and/or predicted proteins belonged to novel bHLH members and were reannotated in this study, that is, NP_001096226.2 (Genbank protein accession), NP_989390.1, NP_001096298.1, NP_001037951.1, NP_001107462.1, NP_001107508.1, NP_001120597.1, NP_001120597.1, XP_002931994.1, XP_002932187.1, XP_002933181.1, XP_002934026.1, XP_002934312.1, XP_002935013.1, XP_002935182.1, XP_002935886.1, XP_002935887.1, XP_002936042.1, XP_002937330.1, XP_002937913.1, XP_002938491.1, XP_002938497.1, XP_002938975.1, XP_002939165.1, XP_002940290.1, XP_002940370.1, XP_002941575.1, XP_002942929.1, XP_002943245.1, XP_002944430.1, XP_002944506.1, XP_002944648.1, XP_002944649.1, and XP_002939654.1. 

**Figure 1:**
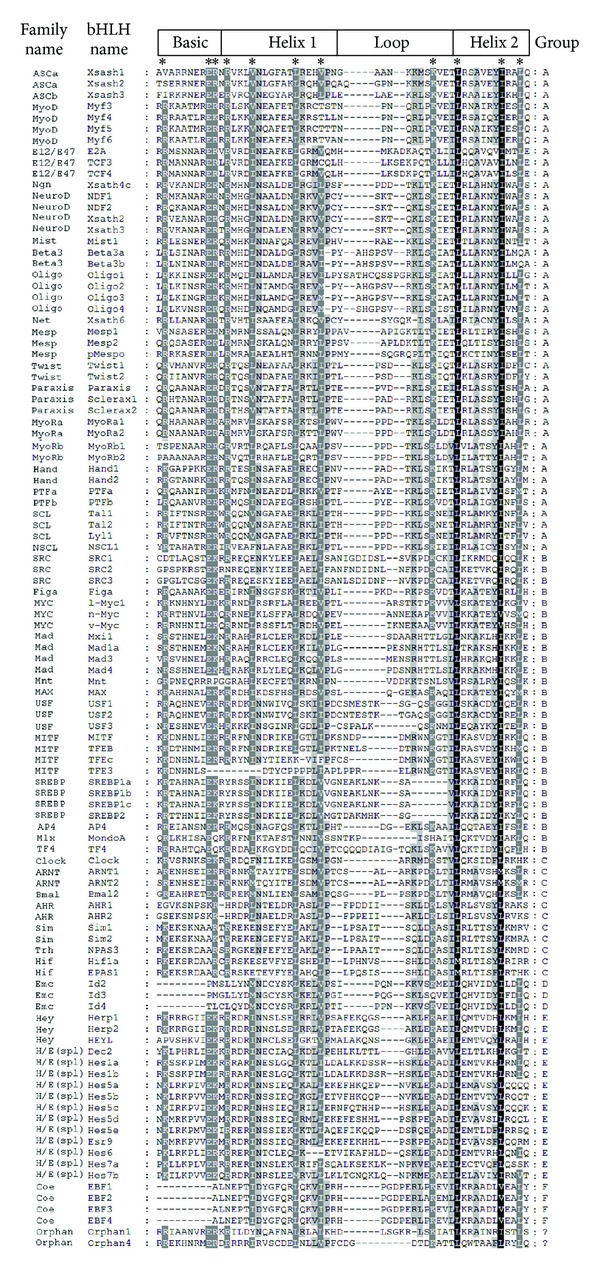
Alignment of 105 *Xenopus tropicalis* bHLH domains. Designation of basic, helix 1, loop, and helix 2 follows Ferre-D'Amare et al. [39–43] and bHLH domains were shaded using GeneDoc. Family and bHLH protein names and high-order groups were organized according to Table 1 in the paper of Ledent et al. [4]. Highly conserved sites are shaded in black and indicated with asterisks on the top.

In total, 105 putative *Xenopus tropicalis* bHLH protein sequences were identified with the BLASTP, TBLASTN, and PSI-BLAST searches and manual examination of the 19 conserved amino acid sites (Table 1, Figure 1). Among these putative bHLH protein sequences, most of these hypothetical proteins were newly produced in the *Xenopus tropicalis* genome project. We further identified and verified these hypothetical proteins with corresponding EST sequences obtained by TBLASTN searches against the expressed sequence database (data not shown).

In summary, two proteins identified belonging to none of these groups were classified as “orphans,” while the other 103 bHLH members belonged to 43 families with 46, 26, 11, 3, 15, and 4 bHLH members in the corresponding high-order groups A, B, C, D, E, and F, respectively. Figure 1 showed the domain alignment of 105 *Xenopus tropicalis* bHLH proteins. In addition, the members of Delilah and Mist families were not found in this research.

### 3.2. Phylogenetic Analyses and Identification of Putative bHLH Proteins

Phylogenetic trees of MLE and BI showed the diversity of the frog bHLH transcription factor family. All of the data of phylogenetic trees for *Xenopus tropicalis* bHLH proteins are available upon request. The topologies of these two inference methods agreed well with each other (Table 1). It was found that both human and frog proteomes have a number of lineage-specific bHLH families and their members. For example, in the *Xenopus tropicalis* proteomes, no orthologous genes for human *TF12*, *Hath1*, *Hath4a*,* Hath4b*, *Hath5*, and* Id1* could be found in the present research. However, the *Xenopus tropicalis* proteomes also have multiple orthologous genes corresponding to one human gene, such as *SREBP1a*, *SREBP1b*, and* SREBP1c* (orthologous genes of human *SREBP1*); *Hes1a* and *Hes1b* (orthologous genes of human *Hes1*); *Hes6a* and *Hes6b* (orthologous genes of human *Hes6*); *Hes5a*, *Hes5b*, *Hes5c*, *Hes5d*, *Hes5e*, and* Esr9* (orthologous genes of human *Hes5*).

### 3.3. Enriched Functional GO Annotations

Gene ontology (GO) annotations including biological process (BP), molecular function (MF), and cellular component (CC) were downloaded and investigated from the gene ontology database (http://www.geneontology.org/), and the genes were grouped according to their GO hierarchy annotations. To explore functional properties and identify groups of genes coding for proteins with similar function or with participation in common regulatory pathways, all of the retrieved putative bHLH genes were grouped and functionally classified and enriched according to available GO annotations, information from curated pathways, and known protein interactions. In the present work, the 105 frog bHLH genes were grouped into 7 supergroups according to Ledent et al. [4] and Simionato et al. [6] to get available GO annotations and their enrichment by categories (cutoff of *P* ≤ 0.05). With gene accessions, protein accessions, and the other eligible sequence information in DAVID Bioinformatics Database [32] for *Xenopus tropicalis* bHLH transcription factors, we retrieved all of the significant GO annotations (cutoff of *P* ≤ 0.05). There were 96 genes fitting the record of DAVID Bioinformatics Database [31, 32] and these genes obtained significant GO annotations, while the other nine genes did not get significant GO annotation and were discarded (mainly group D, F, and Orphans; Table 2).

Among the genes, more than half were annotated as exhibiting “transcription regulator activity” and/or “regulation of transcription” or similar terms related to DNA-dependent regulation of transcription, DNA binding, or regulation of RNA metabolic process in the BP and MF categories. There were three significant KEEG pathways, that is, circadian rhythm (KEGG ID: 480089074, *P* value 0.0039), TGF-beta signaling pathway (KEGG Id: 480089058, *P* value 0.0024), and Notch signaling pathway (KEGG Id: 480089056, *P* value 0.046), and few significant GO terms for bHLH genes identified in the CC category for *Xenopus tropicalis* bHLH proteins in DAVID Bioinformatics Database [32]. In the BP category, a total of 47.83% of the significant GO annotations were annotated as transcription and transcription (factor) activity and/or regulation of transcription, while 28.26% of GO annotations were connected to muscle cell development or differentiation and 26.09% of GO annotations were related to negative regulation of cellular biosynthetic or macromolecular metabolic processes. Several genes in the BP category were associated with neural tube development, floor plate development, sensory organ development, chordate embryonic development, hormone receptor binding, and so forth. In the MF category, 56.25% of GO annotations were connected to transcription factor binding or transcription regulator activity, while 3 out of 16 of the GO annotations were related to DNA binding.

DNA binding, protein dimerization, and transcription coactivator activity are important functional activities of bHLH domains. The DNA binding activity of bHLH proteins is mainly determined by the basic region [2]. Site-directed mutagenesis experiments and the crystal structure studies of bHLH proteins showed that the Glu-9/Arg-12 pair forms the CANNTG recognition motif, the critical Glu-9 contacts the first CA in the DNA-binding motif, and the role of Arg-12 is to fix and stabilize the position of the Glu-9 [35–38, 47]. To further understand the functions of *Xenopus tropicalis* bHLH genes as a whole, we collected GO enrichment data on the 105 *Xenopus tropicalis* bHLH genes with significant hypergeometric *P* values. Among all of the GO terms, 65 significant GO terms (*P* ≤ 0.05) were identified showing key cellular components, molecular functions, biological processes, and KEGG pathways for the 105 *Xenopus tropicalis* bHLH genes (Table 2). Muscle organ development, embryonic development ending in birth or egg hatching, chordate embryonic development, sensory organ development, neural tube development, camera-type eye development and eye development, floor plate development, and muscle fiber and tissue development have high frequencies when taking no account of the frequent GO term categories of transcriptional factors such as (negative) regulation of transcription and regulation of metabolism and biosynthetic processes. It has been well known that the bHLH genes in various groups have special recognition motifs of DNA-binding sites such as E-box and G-box. So, how about the gene functions of each group? To explore these issues, we calculated the hypergeometric distribution enrichment score of gene molecular functions from group A to group F based on GO annotations of GO term categories including biological process, molecular function, cellular component, KEGG pathways, and other key words. However, only significant enriched annotations (cut off *P* ≤ 0.05) in deeper layers (sublayers) are shown in Table 2. GO statistics analyzed with a brief summary of subtypes describing each subgroup are also listed in Table 2.

Our analysis focused on significant GO terms for all of the whole *Xenopus tropicalis* bHLH gene family and for each subgroup (Table 2). We found that each subgroup (except for D and F with few members identified) of bHLH transcription factors has its own specific GO term categories (Table 2), when common GO terms of transcription such as transcription regulator activity, regulation of transcription, and DNA binding and protein dimerization activity are discounted. Group A is characterized with muscle organ development such as (striated) muscle cell differentiation and development, (skeletal) muscle fiber development, (extraocular) skeletal muscle tissue development, and striated muscle and pharyngeal muscle development. In addition, digestive system development, pharynx development, and sensory organ development are also included in this group (Table 2). The functions of bHLH members of group B and group C are mainly composed of transcription, transcription regulator activity, and regulation of transcription. However, group B is different from group C with some GO terms such as transcription coactivator activity, transcription cofactor activity, and (nuclear) hormone receptor binding (Table 2). Group E is composed of some functionally diversified transcription regulators whose GO terms are enriched in many aspects of transcription, such as transcription regulator activity, (negative) regulation of transcription, (negative) regulation of RNA metabolic process, (negative) regulation of transcription from RNA polymerase II promoter, (negative) regulation of nucleobase, nucleoside, and nucleotide and nucleic acid metabolic process, (negative) regulation of biosynthetic process, DNA binding, and protein heterodimerization activity. There are some special GO terms in group E, such as chordate embryonic development, floor plate development, neural tube development, anterior/posterior pattern formation, and (negative) regulation of muscle development (Table 2). KEGG terms, like TGF-beta signaling pathway and Notch signaling pathway, also provide key annotations and insights for bHLH members in group E.

### 3.4. Pathways Analysis

We could identify and select significantly enriched gene ontology terms and pathways using DAVID [31, 32] and KOBAS [33–35] in the present study. We selected functional categories that were more likely to be biologically meaningful by calculating the statistical significance of each functional category in the input set of genes versus all annotated genes in the* Xenopus tropicalis* genome. After the GO annotations of *Xenopus tropicalis* bHLH transcription factors with the DAVID Bioinformatics Tools, all of the bHLH transcription factor genes were also subjected to KOBAS analysis (http://kobas.cbi.pku.edu.cn/home.do) and significant pathways were retrieved at the default *P* values. We applied KOBAS to first annotate all of the genes with KO and to then identify both the most frequent and the statistically significantly enriched pathways. With the strict cutoff of FDR ≤ 0.05, KOBAS found statistically significantly enriched pathways in public databases, such as KEEG, Reactome, and PANTHER, as shown in Table 3. Using this threshold, we identified 16 pathways as induced in the *Xenopus tropicalis* genomic gene samples (Table 3). Among these pathways, 11 pathways were from KEEG database, while six pathways were at the significant level of *P* ≤ 0.05. Interestingly, four of the main central cell signaling systems, that is, Notch signaling pathway, Wnt signaling pathway, TGF-beta signaling pathway, and MAPK signaling pathway, were identified. There were two most significant components related to Notch signaling pathway (corrected *P* value 0.0024084 and 0.0150668) and circadian clock and/or circadian rhythm regulation (corrected *P* value 0.0001219 and 0.0398896), respectively. The Jak-STAT signaling pathway, which is regarded as one of the central cell signaling systemS for muscle development, was identified too. It was the same case that many bHLH proteins were enriched in TGF-beta signaling pathway and Notch signaling pathway as annotated using DAVID Bioinformatics Resources. Furthermore, many interesting pathways were also identified as significantly, such as ErbB signaling pathway, Fanconi anemia pathway, and herpes simplex infection.

### 3.5. Protein Interaction Network

**Figure 2:**
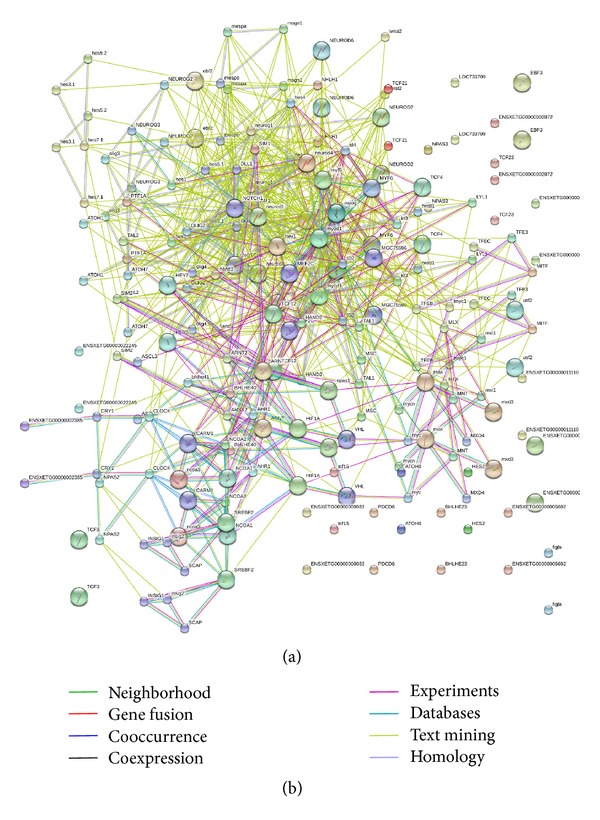
STRING mapping profiles of protein interaction network (PIN) representing bHLH transcription factor protein interactions. Panel (a) showed the main figure of PIN profile and connectivity of hub proteins and the others. The protein interacting gene products are marked in blue and green lines. There are totally 68 hub proteins identified and many hub proteins created a tight network or a functional module within their protein families. Panel (b) magnified the implication of different connective lines with different data sources in the main figure.

To identify putative functional units that consist of proteins coded by the differentially expressed genes, direct and indirect interactions between these proteins were derived using the STRING search tool, which creates PIN files based on previously reported interactions between proteins. Based on 93 bHLH proteins and their 10 predicted functional partners (CARM1, INSIG2, MEF2C, VHL, INSIG1, MGC75596, NOTCH1, DLL1, and SCAP; relevant coefficient ≥ 0.967) in *Xenopus tropicalis* genomic databases, large PIN files were derived and investigated for the presence of hub proteins defined as proteins with at least five interactions to other proteins (Figure 2). Altogether, 68 hub proteins were identified (i.e., MESPA, MESPB, MSGN1, EBF2, NEUROD6, NEUROD4, NEUROD2, NEUROD1, NEUROG1, NEUROG3, NHLH1, TCF21, TCF12, TCF4, TFEB, TFE3, HES4, HES5.1, HES7.1, HES1, DLL1, SIM1, SIM2, ID2, ID3, ID4, MYF6, MYF5, MYOG, MYOD1, NOTCH1, OLIG2, OLIG3, OLIG4, LYL1, HEY1, HEY2, TWIST1, HAND1, HAND2, MEF2C, MGC75596, MLX, MXI1, MAX, LMYC1, MNT, MYC, PTF1A, TAL1, MSC, TAL2, ATOH1, ATOH7, ARNT, ARNT2, AHR1, BHLHE40, BHLHE41, HIF1A, VHL, CLOCK, EPAS1, CARM1, NCOA1, NCOA2, NCOA3, and SREBF2; it should be noted that there are some aliases of bHLH proteins existing in the public databases). Among all proteins in the STRING databases, those were core-connected and had higher expression in many experimental data in the regulatory interaction network (Figure 2).

Interestingly, many hub proteins created a tight network or a functional module within their protein families, such as NEUROD6, NEUROD4, NEUROD2, NEUROD1, NEUROG1, NEUROG3, HES4, HES5.1, HES7.1, HES1, ID2, ID3, ID4, MYF6, MYF5, MYOG, MYOD1, MLX, MXI1, MAX, MITF, and MNT, which are all involved in the same or similar cellular machinery components and/or genetic functions (Figure 2). 

## 4. Concluding Remarks

In this research, we have identified 105 bHLH domains and their protein sequences in the *Xenopus tropicalis* genome databases by TBLASTN, BLASTP, and PSI-BLAST searches with the 45 representative bHLH domains as query sequences. Among these bHLH members, 34 hypothetical proteins, such as LOC100124777, were newly annotated by computational analysis and verified by EST searching in this research. These uncharacterized putative bHLH proteins may be novel transcription factors, which need further validation. The prediction of *Xenopus tropicalis* bHLH transcriptional factors will be very useful for the experiment identifying novel bHLH transcription factors and the construction of transcriptional regulatory network of *Xenopus tropicalis*. Through phylogenetic analyses of the *Xenopus tropicalis* bHLH protein domains with human bHLH orthologous protein sequences, we assigned the 105 *Xenopus tropicalis* bHLH genes to 43 families and two orphan genes according to the 45 defined bHLH families [3, 11]. Two families, for example, Mist and Delilah, were not found in the study. 

Further analysis of the *Xenopus tropicalis* bHLH transcription factors and their functional properties showed that 96 out of 105 bHLH genes could be annotated and only four supergroups' GO enrichment by categories were available [4]. GO enrichment statistics showed 65 significant GO annotations of biological processes and molecular functions counted in frequency. Besides common GO term categories of bHLH transcriptional factors, a large number of *Xenopus tropicalis* bHLH genes play significant role in muscle and organ development, chordate and neural development, floor plate and eye development, and so forth [39–43, 48–55]. Moreover, as the group analysis results described, different groups of proteins have their special gene functions when taking no account of the common GO term categories. The trends of the gene function enrichment may be led by their DNA-binding specificity [51–55]. Therefore, the biology function of the uncharacterized genes or proteins can be predicted through the function GO annotation of the group analysis. To explore the functional pathways, regulatory gene networks and/or related gene groups coding for *Xenopus tropicalis* bHLH proteins, the identified bHLH genes were put into the databases KOBAS and STRING to get the signaling information of pathways and protein interaction networks according to available public databases and known protein interactions. From the KOBAS genomic annotation and pathway analysis, we identified 16 pathways in the *Xenopus tropicalis* genome. From the STRING interaction analysis, 68 hub proteins were identified and many hub proteins created a tight network or a functional module within their protein families.

The present research deepens our knowledge of frog bHLH transcription factors and provides a solid framework for further research on the functional and evolutionary aspects of *Xenopus tropicalis* bHLH transcription factors.

## Figures and Tables

**Table 1:** Information of the *Xenopus tropicalis* 105 bHLH transcription factors.

bHLH family	Gene name	*Homo sapiens* orthologous gene			Protein accession^c^	Genome contig^d^
		Name	MLE bootstrap value (%)^a^	BI posterior probability (%)^b^		
ASCa	*Xsash1 *	*Hash1 (ASCL1) *	89	99	XP_002944648.1	NW_003169609.1
ASCa	*Xsash2 *	*Hash2 *	n/m*	99	XP_002940290.1	NW_003163913.1
ASCb	*Xsash3 *	*Hash3 (ASCL3) *	90	100	XP_002940370.1	NW_003163927.1
MyoD	*Myf3 *	*Myf3 *	96	94	NP_988972.1	NW_003166075.1
MyoD	*Myf4 *	*Myf4 *	94	100	NP_001016725.1	NW_003163495.1
MyoD	*Myf5 *	*Myf5 *	n/m	76	NP_988932.1	NW_003163331.1
MyoD	*Myf6 *	*Myf6 *	82	95	NP_001017160.1	NW_003163331.1
E12/E47	*E2A *	*E2A *	99	53	NP_001093743.1	NW_003163736.1
E12/E47	*TCF3 *	*TCF3 *	76	88	XP_002940299.1	NW_003163915.1
E12/E47	*TCF4 *	*TCF4 *	76	n/m*	NP_001096226.2	NW_003163423.1
Ngn	*Xsath4c *	*Hath4c *	83	78	NP_001116895.1	NW_003163503.1
NeuroD	*NDF1 (neurod1) *	*NDF1 (NEUROD1) *	n/m	n/m*	NP_001090868.1	NW_003163341.1
NeuroD	*NDF2 *	*NDF2 (NEUROD2) *	65	63	NP_001072486.1	NW_003163936.1
NeuroD	*Xsath2 *	*Hath2 *	79	80	NP_001072273.1	NW_003163914.1
NeuroD	*Xsath3 *	*Hath3 *	97	99	NP_001124513.1	NW_003163487.1
Mist1	*Mist1 *	*Mist1 *	99	100	XP_002931994.1	NW_003163340.1
Beta3	*Beta3a *	*Beta3a *	70	53	XP_002944506.1	NW_003167409.1
Beta3	*Beta3b *	*Beta3b *	77	94	NP_001072933.1	NW_003163515.1
Oligo	*Oligo1 *	*Oligo1 *	97	100	XP_002938497.1	NW_003163700.1
Oligo	*Oligo2 *	*Oligo2 *	76	73	XP_002938491.1	NW_003163700.1
Oligo	*Oligo3 *	*Oligo3 *	83	90	NP_001008191.1	NW_003163713.1
Oligo	*Oligo4 *	*Oligo1 Oligo2 * *Oligo3 *	n/m	n/m	NP_001039180.1	NW_003163795.1
Net	*Xsath6 *	*Hath6 *	100	100	XP_002937330.1	NW_003163606.1
Mesp	*Mesp1 *	*Mesp1 Mesp2 * *pMesp1 *	n/m	n/m	NP_001039184.1	NW_003163348.1
Mesp	*Mesp2 *	*Mesp1 Mesp2 * *pMesp1 *	n/m	n/m	NP_001016653.1	NW_003163348.1
Mesp	*pMespo *	*pMesp1 *	99	100	NP_001039104.1	NW_003163426.1
Twist	*Twist1 *	*Twist1 *	91	83	NP_989415.1	NW_003163378.1
Twist	*Twist2 *	*Twist2 *	98	100	NP_001096679.1	NW_003163487.1
Paraxis	*Paraxis *	*Paraxis *	62	83	NP_001016506.1	NW_003165117.1
Paraxis	*Sclerax*1	*Sclerax *	96	99	XP_002942929.1	NW_003164455.1
Paraxis	*Sclerax*2	*Sclerax *	74	59	XP_002937913.1	NW_003163647.1
MyoRa	*MyoRa1 *	*MyoRa1 *	63	60	NP_001096235.1	NW_003163586.1
MyoRa	*MyoRa2 *	*MyoRa2 *	n/m	62	NP_001103518.1	NW_003163498.1
MyoRb	*MyoRb1 *	*MyoRb1 *	78	94	GNOMON∣93674.p^e^ (*ab initio* protein)	NW_003164157.1
MyoRb	*MyoRb2 *	*MyoRb2 *	55	95	GNOMON∣522504.p^e^ (*ab initio* protein)	NW_003163470.1
Hand	*Hand1 *	*Hand1 *	94	100	NP_001016743.1	NW_003163350.1
Hand	*Hand2 *	*Hand2 *	99	55	NP_001093695.1	NW_003163380.1
PTFa	*PTFa *	*PTFa *	99	100	NP_001095279.1	NW_003163378.1
PTFb	*PTFb *	*PTFb *	91	100	XP_002933181.1	NW_003163373.1
SCL	*Tal1 *	*Tal1 *	77	62	NP_001135468.1	NW_003163327.1
SCL	*Tal2 *	*Tal2 *	72	76	XP_002934026.1	NW_003163404.1
SCL	*Lyl1 *	*Lyl1 *	86	97	XP_002939165.1	NW_003163774.1
NSCL	*NSCL1 *	*NSCL1 *	99	100	XP_002937307.1	NW_003163605.1
SRC	*SRC1 *	*SRC1 *	82	97	NP_001106383.1	NW_003163796.1
SRC	*SRC2 *	*SRC2 *	97	100	NP_001135631.1	NW_003163586.1
SRC	*SRC3 *	*SRC3 *	80	97	XP_002933204.1	NW_003163374.1
Fig*α*	*Fig*α**	*Fig*α**	92	100	NP_001016342.1	NW_003163469.1
MYC	*l-Myc *	*L-Myc *	71	65	NP_001011144.1	NW_003164143.1
MYC	*n-Myc *	*n-Myc *	n/m	98	NP_989390.1	NW_003163721.1
MYC	*v-Myc *	*v-Myc *	91	99	NP_001006874.1	NW_003163866.1
Mad	*Mxi1 *	*Mxi1 *	85	97	NP_001008129	NW_003180496.1 NW_003163820.1
Mad	*Mad1 *	*Mad1 *	n/m	88	NP_001072228.1	NW_003163469.1
Mad	*Mad3 *	*Mad3 *	99	100	NP_001017299.1	NW_003163577.1
Mad	*Mad4 *	*Mad4 *	89	100	NP_001096239.1	NW_003164437.1
Mnt	*Mnt *	*Mnt *	n/m	97	NP_001135494.1	NW_003163468.1
MAX	*MAX *	*MAX *	90	100	NP_001008208.1	NW_003163599.1
USF	*USF1 *	*USF1 *	92	99	NP_001096236.1	NW_003168160.1
USF	*USF2 *	*USF2 *	n/m	60	NP_001007857.1	NW_003163677.1
USF	*USF3 *	*USF3 *	85	99	NP_001120597.1	NW_003164188.1
MITF	*MITF *	*MITF *	n/m	n/m	NP_001093747.1	NW_003163951.1
MITF	*TFEb *	*TFEb *	84	100	NP_001072648.1	NW_003163367.1
MITF	*TFEc *	*TFEc *	66	99	XP_002935013.1	NW_003163447.1
MITF	*TFE3 *	*TFE3 *	85	78	XP_002944430.1	NW_003166883.1
SREBP	*SREBP1a *	*SREBP1 *	88	99	XP_002935886.1	NW_003163500.1
SREBP	*SREBP1b *	*SREBP1 *	88	99	XP_002935887.1	NW_003163500.1
SREBP	*SREBP1c *	*SREBP1 *	88	99	XP_002944649.1	NW_003169615.1 NW_003163500.1
SREBP	*SREBP2 *	*SREBP2 *	n/m	67	NP_001116910.1	NW_003163395.1
AP4	*AP4 *	*AP4 *	71	98	NP_001123841.1	NW_003163353.1
Mlx	*MondoA *	*MondoA *	89	100	NP_001090682.1	NW_003163637.1
TF4	*TF4 *	*TF4 *	88	100	GNOMON:712044.p^e^ (*ab initio* protein)	NW_003164277.1, NW_003164157.1
Clock	*Clock *	*Clock *	99	100	NP_001122127.1	NW_003163433.1
ARNT	*ARNT1 *	*ARNT1 *	n/m	n/m	NP_001116925.1	NW_003163477.1
ARNT	*ARNT2 *	*ARNT2 *	100	n/m	NP_001093686.1	NW_003163348.1
Bmal	*Bmal2 *	*Bmal2 *	63	100	NP_001096298.1	NW_003164805.1
AHR	*AHR1 *	*AHR1 *	92	99	XP_002933348.1	NW_003163378.1
AHR	*AHR2 *	*AHR2 *	91	100	XP_002935182.1	NW_003163457.1
Sim	*Sim1 *	*Sim1 *	n/m*	98	XP_002932187.1	NW_003163345.1
Sim	*Sim2 *	*Sim2 *	89	99	XP_002941575.1	NW_003164120.1
Trh	*NPAS3 *	*NPAS3 *	n/m	70	NP_001072647.1	NW_003163363.1
HIF	*Hif1*α**	*Hif1*α**	99	n/m	NP_001011165.1	NW_003163817.1
HIF	*EPAS1 *	*EPAS1 *	79	94	NP_001005647.1	NW_003163351.1
Emc	*Id2 *	*Id2 *	78	90	NP_988885.1	NW_003163451.1
Emc	*Id3 *	*Id3 *	79	98	NP_001016271.1	NW_003163432.1
Emc	*Id4 *	*Id4 *	86	54	NP_001004839.1	NW_003163385.1
Hey	*Herp1 *	*Herp1 *	83	97	NP_001007911.1	NW_003163551.1
Hey	*Herp2 *	*Herp2 *	86	92	XP_002936042.1	NW_003163507.1
Hey	*HEYL *	*HEYL *	98	100	XP_002934312.1	NW_003163416.1
H/E(spl)	*Dec2 *	*Dec2 *	99	n/m	NP_001027504.1	NW_003163993.1
H/E(spl)	*Hes1a *	*Hes1 *	n/m	81	NP_001011194.1	NW_003163571.1
H/E(spl)	*Hes1b *	*Hes1 *	n/m	81	NP_988870.1	NW_003163533.1
H/E(spl)	*Hes5a *	*Hes5 *	n/m*	61	NP_001037880.1	NW_003163546.1
H/E(spl)	*Hes5b *	*Hes5 *	n/m*	61	NP_001037974.1	NW_003163546.1
H/E(spl)	*Hes5c *	*Hes5 *	n/m*	100	NP_001039178.1	NW_003163399.1
H/E(spl)	*Hes5d *	*Hes5 *	n/m*	100	NP_001037951.1	NW_003163399.1
H/E(spl)	*Hes5e *	*Hes5 *	n/m*	82	NP_001107462.1	No finding
H/E(spl)	*Esr9 *	*Hes5 *	n/m*	100	NP_001037989.1	NW_003163399.1
H/E(spl)	*Hes6 *	*Hes6 *	n/m	n/m	NP_001072210.1	NW_003163381.1
H/E(spl)	*Hes7a *	*Hes7 *	73	97	NP_001039166.1	NW_003164377.1
H/E(spl)	*Hes7b *	*Hes7 *	86	100	NP_001107508.1	NW_003164377.1
Coe	*EBF1 *	*EBF1 *	n/m	51	XP_002939654.1	NW_003163834.1
Coe	*EBF2 *	*EBF2 *	91	97	NP_989200.1	NW_003163356.1
Coe	*EBF3 *	*EBF3 *	91	66	XP_002932694.1	NW_003163358.1
Coe	*EBF4 *	*EBF4 *	91	66	XP_002932695.1	NW_003163358.1
Orphan	*Orphan1 *	*Orphan1 *	86	100	XP_002938975.1	NW_003163749.1
Orphan	*Orphan4 *	*Orphan4 *	94	100	XP_002943245.1	NW_003164609.1

*Xenopus tropicalis* bHLH genes were named according to their human orthologous genes' names (or common abbreviations) and the referred nomenclature was mainly from the tables and additional tables provided by Ledent et al. [4] and Simionato et al. [6]. Bootstrap values were converted from phylogenetic analyses with human bHLH sequences using BI and MLE algorithm, respectively. MLE bootstrap value^a^ refers to the result from maximum likelihood estimate in phylogenetic analysis, and BI posterior probability^b^ refers to the result from BI in phylogenetic analysis. The numbers in the phylogenetic trees are converted into percentages. ^c^The accession numbers were retrieved from the following resources; this sequence was verified by many EST TBLASTN search hits, such as EG651417.1 and CX503003.2 (EST accession number). These numbered as “NP” were from the RefSeq protein database and those numbered as “XP” were from the Build protein database. Notes in the brackets are also gene symbols according to records in NCBI and Xenbase. All of the bHLH genes are organized in the order of bHLH families manifested in Table 1 of Ledent et al. [4]. The question mark means no matching; mark n/m* means no monophyletic group with single particular orthologous gene sequences, but formed a monophyletic group with two or more orthologous gene sequences of the family; mark n/m denotes the case of lower bootstrap value estimated less than 50%.^e^The accession numbers were retrieved from the *ab initio* protein database.

**Table 2:** GO enrichment by categories of super-groups by DAVID bioinformatics bases with 105 *Xenopus tropicalis* bHLH transcription factors.

Group	Enriched genes	GO term ID	GO category	GO definition	Coherence (%)^a^	*P* value
A	43	GO:0030528	MF	Transcription regulator activity	100	2.50*E* − 27
		GO:0045449	BP	Regulation of transcription	100	7.60*E* − 22
		GO:0007517	BP	Muscle organ development	15.4	5.50*E* − 05
		GO:0007519	BP	Skeletal muscle tissue development	7.7	1.30*E* − 02
		GO:0055123	BP	Digestive system development	7.7	1.30*E* − 02
		GO:0014706	BP	Striated muscle tissue development	7.7	1.30*E* − 02
		GO:0043282	BP	Pharyngeal muscle development	7.7	1.30*E* − 02
		GO:0002074	BP	Extraocular skeletal muscle development	7.7	1.30*E* − 02
		GO:0048741	BP	Skeletal muscle fiber development	7.7	1.30*E* − 02
		GO:0048747	BP	Muscle fiber development	7.7	1.30*E* − 02
		GO:0060538	BP	Skeletal muscle organ development	7.7	1.30*E* − 02
		GO:0060465	BP	Pharynx development	7.7	1.30*E* − 02
		GO:0007423	BP	Sensory organ development	11.5	1.70*E* − 02
		GO:0042692	BP	Muscle cell differentiation	7.7	2.00*E* − 02
		GO:0060537	BP	Muscle tissue development	7.7	2.00*E* − 02
		GO:0051146	BP	Striated muscle cell differentiation	7.7	2.00*E* − 02
		GO:0055002	BP	Striated muscle cell development	7.7	2.00*E* − 02
		GO:0055001	BP	Muscle cell development	7.7	2.00*E* − 02
		GO:0003677	MF	DNA binding	26.9	8.70*E* − 02

B	267	GO:0030528	MF	Transcription regulator activity	100	8.10*E* − 20
		GO:0045449	BP	Regulation of transcription	100	6.90*E* − 16
		GO:0035257	MF	Nuclear hormone receptor binding	10.5	1.20*E* − 02
		GO:0051427	MF	Hormone receptor binding	10.5	1.60*E* − 02
		GO:0003713	MF	Transcription coactivator activity	10.5	2.00*E* − 02
		GO:0003712	MF	Transcription cofactor activity	10.5	3.10*E* − 02
		GO:0008134	MF	Transcription factor binding	10.5	6.90*E* − 02
		GO:0006355	BP	Regulation of transcription, DNA-dependent	26.3	9.70*E* − 02

C	11	GO:0006350	BP	Transcription	100	1.40*E* − 07
		GO:0030528	MF	Transcription regulator activity	100	4.70*E* − 07
		GO:0006355	BP	Regulation of transcription, DNA-dependent	100	9.50*E* − 07
		GO:0051252	BP	Regulation of RNA metabolic process	100	1.00*E* − 06
		GO:0003677	MF	DNA binding	100	4.10*E* − 06
		GO:0045449	BP	Regulation of transcription	100	9.30*E* − 06
		GO:0003700	MF	Transcription factor activity	71.4	1.70*E* − 04
		KEGG_Id:480089074	KEGG pathway	Circadian rhythm	28.6	3.90*E* − 03

D	3	None	None	None	None	None

E	15	GO:0030528	MF	Transcription regulator activity	100	1.30*E* − 16
		GO:0045449	BP	Regulation of transcription	100	2.40*E* − 13
		GO:0006350	BP	Transcription	68.8	7.90*E* − 09
		GO:0003677	MF	DNA binding	75	1.10*E* − 07
		GO:0006355	BP	Regulation of transcription, DNA-dependent	68.8	1.70*E* − 07
		GO:0051252	BP	Regulation of RNA metabolic process	68.8	1.90*E* − 07
		GO:0016564	MF	Transcription repressor activity	31.2	2.90*E* − 07
		GO:0000122	BP	Negative regulation of transcription from RNA polymerase II promoter	25	1.80*E* − 05
		GO:0009792	BP	Embryonic development ending in birth or egg hatching	25	7.50*E* − 05
		GO:0043009	BP	Chordate embryonic development	25	7.50*E* − 05
		GO:0045892	BP	Negative regulation of transcription, DNA-dependent	25	1.60*E* − 04
		GO:0051253	BP	Negative regulation of RNA metabolic process	25	1.80*E* − 04
		GO:0046982	MF	Protein heterodimerization activity	18.8	2.10*E* − 04
		GO:0021915	BP	Neural tube development	18.8	2.20*E* − 04
		GO:0016481	BP	Negative regulation of transcription	25	2.80*E* − 04
		GO:0007219	BP	Notch signaling pathway	18.8	3.10*E* − 04
		GO:0051172	BP	Negative regulation of nitrogen compound metabolic process	25	3.70*E* − 04
		GO:0045934	BP	Negative regulation of nucleobase, nucleoside, nucleotide and nucleic acid metabolic process	25	3.70*E* − 04
		GO:0031327	BP	Negative regulation of cellular biosynthetic process	25	4.60*E* − 04
		GO:0010558	BP	Negative regulation of macromolecule biosynthetic process	25	4.60*E* − 04
		GO:0009890	BP	Negative regulation of biosynthetic process	25	5.00*E* − 04
		GO:0010629	BP	Negative regulation of gene expression	25	5.70*E* − 04
		GO:0006357	BP	Regulation of transcription from RNA polymerase II promoter	25	8.90*E* − 04
		GO:0010605	BP	Negative regulation of macromolecule metabolic process	25	9.50*E* − 04
		KEGG_Id:480089058	KEGG pathway	TGF-beta signaling pathway	18.8	2.40*E* − 03
		GO:0033504	BP	Floor plate development	12.5	7.90*E* − 03
		GO:0046983	MF	Protein dimerization activity	18.8	9.20*E* − 03
		GO:0048635	BP	Negative regulation of muscle development	12.5	1.20*E* − 02
		GO:0048634	BP	Regulation of muscle development	12.5	1.60*E* − 02
		KEGG_Id:480089056	KEGG pathway	Notch signaling pathway	12.5	4.60*E* − 02

F	4	None	None	None	None	None

Orphan	2	None	None	None	None	None

All GO annotations terms in the table were from gene ontology database (http://www.geneontology.org/). GO annotations included every layer of biological process, molecular function, cellular component category, and KEGG pathway. When a GO term and its sublayer GO are both enriched in a group significantly, only deeper layer GO annotation is shown in the table. BP: biological process; MF: molecular function. The above table showed the GO annotations enriched significantly (*P* < 0.05) in each group. ^a^GO coherence of each group, measured as the percentage of genes in group covered by the GO category.

**Table 3:** Significant pathways identified by KOBAS with 93 *Xenopus tropicalis* bHLH transcription factors.

Term	Pathway database	Database ID	Sample gene number	Background number	*P* value	Corrected *P* value	Genes
Circadian rhythm: mammal	KEGG	xtr04710	3	21	1.10*E* − 05	0.0001219	XSBmal2; XSDec2; XSClock
TGF-beta signaling pathway	KEGG	xtr04350	4	73	1.52*E* − 05	0.0001219	XSId3; XSId2; XSId4; XSnMyc
Notch signaling pathway	PANTHER	P00045	2	5	0.0004516	0.0024084	XSHes1a; XSHes1b; XSHerp1
Notch signaling pathway	KEGG	xtr04330	2	43	0.0037667	0.0150668	XSHes1a; XSHes1b; XSHes5a
Developmental biology	Reactome	None	2	106	0.0145401	0.0398896	XSHes1a; XSHes1b; XSNDF1; XSNDF2
Circadian clock	Reactome	None	1	8	0.0149586	0.0398896	XSClock
Herpes simplex infection	KEGG	xtr05168	2	128	0.0304156	0.0695215	XSBmal2; XSClock
MAPK signaling pathway	KEGG	xtr04010	2	220	0.0802242	0.1604484	XSMAX; XSnMyc
Fanconi anemia pathway	KEGG	xtr03460	1	51	0.1044307	0.1856547	XSHes1a; XSHes1b
ErbB signaling pathway	KEGG	xtr04012	1	70	0.1406256	0.2250009	XSnMyc
Melanogenesis	KEGG	xtr04916	1	86	0.1700261	0.2386888	XSMITF
Jak-STAT signaling pathway	KEGG	xtr04630	1	91	0.1790166	0.2386888	XSnMyc
Metabolism	Reactome	None	2	458	0.206835	0.2544026	XSSRC2; XSSRC1
Cell cycle	KEGG	xtr04110	1	116	0.2226023	0.2544026	XSnMyc
Wnt signaling pathway	KEGG	xtr04310	1	131	0.2476916	0.2642044	XSnMyc
Wnt signaling pathway	PANTHER	P00057	1	37	0.2756772	0.2756772	XSvMyc
